# Supplementary material for: An Emerging Role for Sigma-1 Receptors in the Treatment of Developmental and Epileptic Encephalopathies
Source: Int J Mol Sci. 2021 Aug 5;22(16):8416. doi: 10.3390/ijms22168416 (PMC8395113; doi:10.3390/ijms22168416)
Supplement: Supplementary file 1 [file ijms-22-08416-s001.zip › ijms-1285045-supplementary.pdf]

Table S1. Clinical outcomes of fenfluramine treatment for developmental and epileptic encephalopathies.

Study	Treatment Duration	Endpoint	Results	Reference
**Dravet syndrome, phase 3 RCTs[a,b]**				
Study 1 (N = 119)	14 weeks T + M	Change in MCSF from placebo to T + M	0.7 mg/kg/day FFA: -62.3% (p < 0.0001)<br>0.2 mg/kg/day FFA: -32.4% (p = 0.0209)	Lagae 2020 [8]
Study 2 (N = 87)	15 weeks T + M	Change in mean MCSF from placebo to T + M	0.4 mg/kg/day FFA[c]: -54.0% (p < 0.001)	Nabbout 2020 [9]
Study 3 (N = 143)	14 weeks T + M	Change in mean MCSF from placebo	0.7 mg/kg/day FFA: -64.8% (p < 0.0001)<br>0.2 mg/kg/day FFA: -49.9% (p < 0.0001)	Sullivan 2020 [10]
**Dravet syndrome, OLE**	Median: 256 days (range: 46−634)	Median change in MCSF from baseline in the core study	-66.8% (p < 0.001)	Sullivan 2020 [11]
**Dravet syndrome, post hoc analyses**				
BRIEF®[c]	14 weeks T + M	Change in BRIEF® indexes from baseline to T + M compared to placebo	Improvement in executive function as measured by the Behavioral Regulation Index at 0.7 mg/kg/day FFA (p = 0.0117) and 0.2 mg/kg/day FFA (p = 0.0185)<br>Improvement in Global Executive Composite at 0.7 mg/kg/day FFA (p = 0.0245)	Lagae 2020 [8]
BRIEF®2[c] (N = 58)	1 year	Change in BRIEF®2 indexes from baseline to Year 1	Improvement in executive function as measured by Emotion Regulation Index and Cognitive Regulation Index in 22% and 24% of patients, respectively, with MCSF reduction ≥50% from core study baseline (vs. 0% in placebo; p = 0.002 and p = 0.001)	Bishop 2021 [12]
SUDEP	Up to 32 years	Incidence of SUDEP in Dravet syndrome before and during treatment with FFA in US and EU EAP, phase 3 studies, 2 Belgian cohorts	Cooper et al., 2016 (N = 100; historical controls, no FFA): 9.32 deaths/1000 patient-years<br>No FFA (N = 366): 11.7 deaths/patient-year<br>FFA (N = 732): 1.7 deaths/patient-year	Cross 2020 [13]
**LGS RCT[b]**	14 weeks T + M	Change in MCSF from placebo to T + M	0.7 mg/kg/day FFA: -19.9% (p = 0.0013)<br>0.2 mg/kg/day FFA: -10.5% (p = NS)	Knupp 2020 [7]
BRIEF®, RCT[c] (N = 137)	14 weeks T + M	Change in BRIEF® indexes from baseline to T + M compared to placebo	0.7 or 0.2 mg/kg/day FFA improved aspects of executive function as measured by Cognitive Regulation Index in 27% (vs. 13% placebo; p = 0.046) and Global Executive Composite score in 25% (vs. 11% placebo; p = 0.034)	Bishop 2021 [14]
**CDD IIS (N = 6)[b]**	≥14 weeks T + M	Median change in seizure frequency from baseline	GTC (n = 5): -90%<br>TS (n = 2): -55%	Devinsky 2021 [15]
**Sunflower syndrome IIS[b] (N = 5)**	1 (n = 1) or 2 months (n = 4)	Median change in MCSF from pretreatment baseline	-74%	Thiele 2020 [16]
**Sunflower syndrome IIS[b,c]** (N = 9 completed study)	3 months (mean, 47.4 days)	≥30% reduction in hand-waving episodes<br>EEG<br>Mean full-scale IQ score	8/9 (89%); 6/9 (67%) experienced ≥70% reduction in hand-waving episodes. In several patients:<br>Reduction in epileptiform activity<br>Resolution of photo-paroxysmal response; slight increase (p = 0.06)	Geenen, 2021 [17]

[a]Stiripentol was an inclusion criterion for Study 2 and an exclusion criterion for Studies 1 and 3. FFA doses were adjusted for a known pharmacological effect with stiripentol.

[b]Seizure outcomes.

[c]Non-seizure outcomes. BRIEF®, Behavior Rating Inventory of Executive Function (BRIEF® scores mapped to updated BRIEF®2 version); BRIEF®2, Behavior Rating Index of Executive Function Second Edition; CDD, CDKL5 deficiency disorder; FFA, fenfluramine; IIS, investigator-initiated study; GTC, generalized tonic-clonic seizure; LGS, Lennox-Gastaut syndrome; MCSF, monthly convulsive seizure frequency; NS, not statistically significant; OLE, open-label extension; RCT, randomized clinical trial; SUDEP, sudden unexpected death in epilepsy; T + M, titration and maintenance; TS, tonic seizure.

Table S2. Pharmacological and functional targets for fenfluramine and its major metabolite norfenfluramine.

Target	Model	Mechanism	Reference
5-HT releaser	In vitro: Rat brain synaptosomes; *Xenopus* oocytes<br>In vivo: Rat nucleus accumbens	SERT substrate, reverse transporter	Baumann 2014 [4]
**5-HT Receptors**			
5-HT_{1A}	Radioligand binding in rat cerebral cortex	FFA: $K_i = 3.27 \times 10^{-7}$ M<br>nFFA: $K_i = 6.73 \times 10^{-7}$ M	Martin 2020 [22]
5-HT_{1A}, CB1	In vitro: GST fusion recombinant protein binding assays	1. FFA and nFFA activate 5-HT_{1A} to inhibit excitatory NMDA activity (i.e., anticonvulsant)	Rodriguez-Munoz 2018 [24]
5-HT_{2A}, 5-HT_{2C}	In vivo: i.c.v. NMDA injection in mice	2. FFA and nFFA agonist activity at 5-HT_{2A} and 5-HT_{2C} and FFA/nFFA interaction with Sigma1R cooperate to remove Sigma1R/HINT1 coupling with NMDAR, thereby enabling negative control of NMDAR by CaM	
5-HT_{2A}, 5-HT_{2B}, 5-HT_{2C}	Radioligand binding in HEK 293E cells; CHO-K1 cells	FFA: weak agonist ($K_i > 0.7-1.5$ µM)<br>nFFA: more potent agonist ($K_i = 27-267$ nM)	Fitzgerald 2000; Porter 1999 [5, 6]
$5HT_3$	Rat SUDEP model	Fluoxetine: Inhibited seizure-induced respiratory arrest at 5-HT_3 without affecting seizures	Faingold 2016 [25]
5-HT_4, 5-HT_2, 5-HT_7	In vivo mouse DBA-1 model of SUDEP	FFA: Anticonvulsant activity at 5-HT_4, 5-HT_2, 5-HT_7<br>FFA: Inhibited seizure-induced respiratory arrest at 5-HT_4	Faingold 2019 [26]
5-HT_{1D}, 5-HT_{2C}	Zebrafish *scn1Lab-/-* model of Dravet syndrome	FFA-mediated inhibition of epileptiform activity and hyperlocomotion reduced by 5-HT_{1D} and 5-HT_{2C} antagonists	Sourbron 2017 [23]
**Sigma Receptors**			
Sigma (non-selective)	Radioligand binding in guinea pig brain	FFA: $K_i = 2.66 \times 10^{-7}$ M<br>nFFA: $K_i = 2.92 \times 10^{-6}$ M	Martin 2020 [22]
Sigma1R	Assay for Sigma1R activity using the cell-based Sigma1R/BiP dissociation assay	FFA: Potentiated activity of PRE-084 (Sigma1R agonist); e.g., shows positive modulatory activity	Martin 2020 [22]
Sigma1R	Assay for Sigma1R activity using ex vivo vas deferens contraction model	FFA: Potentiated activity of (+)-SKF-10,047 (Sigma1R agonist); e.g., shows positive modulatory activity	Martin 2020 [22]
Sigma1R	Mouse model of dizocilpine-induced amnesia	FFA: Potentiated activity of PRE-084 (Sigma1R agonist) in both spontaneous alternation and passive avoidance tests of learning and memory; e.g., shows positive modulatory activity;<br>all combination effects of FFA and PRE-084 in both tests fully blocked by NE-100 (Sigma1R antagonist)	Martin 2020 [22]
Sigma1R	In vitro: GST fusion recombinant protein binding assays<br>In vivo: i.c.v. NMDA injection in mice	Association of Sigma1R with NR1 subunits inhibited by FFA and nFFA<br>NMDA-induced seizures inhibited by FFA and nFFA by preventing Sigma1R/HINT1 association with NR1	Rodriguez-Munoz 2018 [24]
Sigma1R	Zebrafish *scn1Lab* mutant model of Dravet syndrome	FFA-mediated inhibition of epileptiform activity reduced and FFA-mediated hyperlocomotion completely abolished by Sigma1R agonist PRE-084 in combination with 5-HT_{1D} and 5-HT_{2C} antagonists	Sourbron 2017 [23]
Sigma1R	Zebrafish *scn1a* mutant model of Dravet syndrome	Inhibition of epileptogenic activity by administration of the Sigma1R positive allosteric modulator SOMCL-668	Reported here (**Fig. 3**)
Sigma1R	Sigma1R binding in crude synaptic membrane preparations from rat brain	FFA binding with sub-micromolar affinity	Cagnotto 1994 [27]
**Dendritic Arborization of GABAergic Neurons**			
GABAergic neurons	Zebrafish *scn1lab* mutant	Dendritic branching of GABAergic neurons restored by FFA	Tiraboschi 2020 [28]

Pharmacological or functional activity has not been reported at 5-HT_3, 5-HT_5, 5-HT_6, or 5-HT_7 receptors. 5-HT, serotonin; BiP, immunoglobulin-binding protein; CaM, calmodulin; CB, cannabinoid; CHO, Chinese hamster ovary; FFA, fenfluramine; GABA, γ-aminobutyric acid; GST, glutathione S-transferase; HEK, human embryonic kidney; HINT1, histidine triad nucleotide binding protein 1; i.c.v., intracerebroventricular; nFFA, norfenfluramine; NMDA, N-methyl-D-aspartate; NMDAR, NMDA receptor; NR, NMDA receptor subunit; SERT, serotonin transporter; Sigma1R, sigma-1 receptor; SOMCL-668, positive Sigma1R modulator.
